# Cervical cancer screening: Sharing best practices and addressing common challenges in cervical cancer screening programs

**DOI:** 10.1002/ijc.35220

**Published:** 2024-10-26

**Authors:** Philippe Descamps, Francesc Xavier Bosch Jose, Joseph Monsonego, Ody Neisingh, Lananh Nguyen, Mairead O'Connor

**Affiliations:** ^1^ Co‐Chair, ACCESS Consensus Group, Department of Obstetrics and Gynaecology University Hospital Angers Angers France; ^2^ Co‐founder, Clinical Oncologist and Epidemiologist HPV Information Center (ICO and IARC), HPV World (HPW) Barcelona Spain; ^3^ Gynaecologist‐Oncologist Institut du Col Paris France; ^4^ Independent Consultant and Public Affairs Advisor with extensive working experience at WOMEN Inc. and UN Women, and Member of the European Economic and Social Committee on Behalf of Gender Equality Civil Society Amsterdam The Netherlands; ^5^ Director of Cytopathology and Associate Professor, Department of Laboratory Medicine, Unity Health Toronto University of Toronto Toronto Ontario Canada; ^6^ Research Officer National Screening Service Ireland Dublin Ireland

Dear Editor,

There is a concerning trend of declining or stagnated cervical cancer screening participation in some high‐income countries, which disproportionately affects women from disadvantaged communities.^1^, ^2^ For example, in the United Kingdom, screening participation declined from 78.6% in 2011 to 70.2% in 2021, and in the Netherlands, from 66.2% in 2011 to 45.7% in 2022.^3^, ^4^ This is important as strategies for eliminating cervical cancer, which include high uptake of vaccination, continue to require screening to be deployed for several decades for both unvaccinated and vaccinated individuals.

The reasons behind this suboptimal screening participation are multifaceted, encompassing issues such as lack of awareness, cultural beliefs, geographical and financial barriers, and limited access to healthcare. For younger screen‐eligible cohorts, HPV vaccination may also create a sense of security that may detract from screening participation. Urgent action is imperative to reverse this trend and prevent needless deaths from this largely preventable disease.

Recognizing the urgency, the “Advancing Cervical CancEr ScreeningS” (ACCESS) International Consensus Group was established in 2023 as a coalition of leading clinicians, researchers, patient, and women's advocates to advance women's health by increasing participation in cervical cancer screening.

On 13 March 2024, ACCESS convened a diverse group of senior healthcare professionals, researchers and cervical cancer screening program managers from Europe and North America at a roundtable meeting at the EUropean Research Organization on Genital Infection and Neoplasia (EUROGIN) International Multidisciplinary HPV Congress in Stockholm to discuss the six key high‐level recommendations in the Consensus Group's White Paper “Turning The Tide: Recommendations To Increase Cervical Cancer Screening Among Women Who Are Under‐Screened”^5^:

1. Develop cervical cancer national elimination plans with goals for elimination by a defined date, including ambitious national screening program participation targets.
2. Implement targeted and culturally relevant education, information, and awareness‐raising initiatives, particularly focused on under‐screened women.
3. Improve the accessibility of cervical cancer screening.
4. Support healthcare professionals to increase participation in cervical cancer screening.
5. Encourage and support the creation of national cervical cancer patient advocacy groups and national cervical cancer prevention coalitions.
6. Ensure that health insurance appropriately covers screening in all high‐income countries.

The session provoked discussion around best practices and common challenges within cervical cancer screening programs.

## HPV SELF‐SAMPLING

Offering self‐sampling only to under‐screened women has the potential to increase screening participation.^6^, ^7^ However, during the roundtable discussion, experts discussed the impact of self‐sampling and cautioned against viewing it as a panacea for addressing low participation rates. Real‐world experience was shared from countries with relatively high but declining participation rates, where self‐sampling has already been introduced but has so far not resulted in substantial increases in uptake among under‐screened individuals. One expert predicted that, in the best‐case scenario, around 20% of the population would use self‐sampling if available.

Further, in the largest population‐based real‐world study to date,^8^ a large number of individuals who regularly participated in screening switched from provider‐collected to self‐sampling. This was viewed as concerning given test performance: relative CIN2+ detection with self‐sampling was estimated as low as 76% in comparison to clinician‐collected samples in the same study.^8^ This would mean that almost one in four women with cervical lesions could potentially be missed when using self‐sampling in screening programs.

The speakers emphasized that follow‐up after a positive test result has proven particularly difficult in cases of women using self‐sampling. Self‐sampling was associated with a fourfold higher risk of loss to follow‐up compared to women who were screened by their general practitioner, resulting in missed CIN2+/3+ lesions, potentially reducing overall screening program effectiveness.^9^

Some experts present argued that, regardless of the challenges identified, women should be offered a choice of screening modality, including information on the advantages and disadvantages of self and provider‐collected screening options.

## TARGETED COMMUNICATION STRATEGIES

A second important outcome from the meeting was the sharing of country‐level initiatives that have been successful in addressing screening participation among under‐screened populations. An example mentioned was an equity‐driven project in Ireland focusing on women over 60, which yielded promising results, achieving a 24.9% screening participation rate in this age range through a multidisciplinary approach combining bespoke invitation letters and tailored communication for both women and healthcare professionals.^10^

## SUPPORT FOR PROVIDERS

A number of experts spoke about the importance of providing adequate financial incentives for healthcare professionals charged with providing screening, in particular general practitioners and practice nurses. Given the significant pressures on healthcare professionals, the lack of appropriate compensation was seen as a key factor in providers' coverage targets being missed or their limited engagement in trying to increase participation among eligible patients. Some countries have deployed dedicated education teams to educate sample‐takers. The adoption of new educational platforms such as webinars and e‐learning modules, coupled with the establishment of standardized education protocols, were also cited as key tools.

## DATA INFRASTRUCTURE

Finally, experts highlighted the role of robust IT infrastructure and data management systems. Addressing challenges stemming from data privacy regulations (e.g., the General Data Protection Regulation in the EU) and access to population‐based data, test results, and distribution of screening invitations, is paramount to ensure the effectiveness of screening programs. In the Netherlands, general practitioners are not able to access self‐sampling test results due to personal data privacy laws, which may lead to a higher risk of loss to follow‐up after a positive self‐test result.

It was clear from the roundtable discussion that there is no silver bullet to address the challenges in screening participation. Holistic approaches addressing all factors that contribute to sub‐optimal participation are needed. The meeting highlighted the significant benefits of sharing best practices and innovative approaches through international dialogue and collaboration among stakeholders, including policymakers, screening program managers, healthcare professionals, patient advocacy groups, and representatives of under‐screened populations. Such cooperation is essential to advance towards cervical cancer elimination.

## AUTHOR CONTRIBUTIONS

**Philippe Descamps:** Writing – original draft; writing – review and editing; project administration; supervision; conceptualization; methodology. **Francesc Xavier Bosch Jose:** Writing – review and editing. **Joseph Monsonego:** Writing – review and editing. **Ody Neisingh:** Writing – review and editing. **Lananh Nguyen:** Writing – review and editing. **Mairead O'Connor:** Writing – review and editing.

## CONFLICT OF INTEREST STATEMENT

The work of the ACCESS Consensus Group is supported by Hologic. Hologic has no editorial control over the content produced by the group. Philippe Descamps, Francesc Xavier Bosch Jose, Joseph Monsonego, Ody Neisingh, and Lananh Nguyen reported financial compensation from Hologic for their activities as members of the ACCESS Consensus Group. Mairead O'Connor reported reimbursement for travel, speaking time, and accommodation from the ACCESS Consensus Group (paid by Hologic) for a roundtable discussion event at the European Parliament in January 2024.
